# Malignant Perivascular Epithelioid Cell Tumor of the Esophagus

**DOI:** 10.1155/2012/438505

**Published:** 2012-08-22

**Authors:** Matteo Fassan, Mauro Cassaro, Massimo Vecchiato, Roberto Clemente, Gianmaria Pennelli, Stefano Merigliano, Giuseppe Altavilla

**Affiliations:** ^1^Surgical Pathology and Cytopathology Unit, Department of Medicine (DIMED), University of Padua, 35121 Padua, Italy; ^2^General Oncology Unit, Department of Surgery, Oncology and Gastroenterology (DiSCOG), University of Padua, 35121 Padua, Italy; ^3^Surgery Unit, Department of Surgery, Oncology and Gastroenterology (DiSCOG), University of Padua, 35121 Padua, Italy

## Abstract

Malignant perivascular epithelioid cell tumor (PEComa) is a rare tumor composed of hybrid tumor cells characterized by immunoreactivity for both melanocytic and smooth muscle markers. This paper describes the uncommon esophageal location of an 8 cm PEComa in a 75-year-old Caucasian man who was presented with ingravescent dysphagia. Although PEComas arising within the gastrointestinal tract are exceptional findings, clinicians should not exclude this class of tumors in the diagnostic investigation of a bulky lesion of the esophageal wall.

## 1. Introduction

Originally described by Liebow and Castleman in 1971 [1], perivascular epithelioid cell tumor (PEComa; also referred to as clear cell “sugar” tumor) is a category of mesenchymal tumors of relatively recent acquisition in the World Health Organization (WHO) classification of tumors of soft tissue and bone [2].

Histologically, PEComas are composed by a hybrid tumor cell which is characterized by immunoreactivity for both melanocytic (HMB45 and/or Melan-A) and smooth muscle (*α*-smooth muscle actin and/or desmin) markers [3–8].

In the English literature, about hundred cases (in almost every body site) have been reported; they often occur in middle-aged patients, with a female predominance (female-to-male ratio nearly to 7 : 1) [2–4]. In the PEComa family of tumors some other nosological entities have been included: angiomyolipoma, clear cell “sugar” tumor of the lung, lymphangioleiomyomatosis, clear cell myomelanocytic tumor of the falciform ligament/ligamentum teres, and unusual clear cell tumors of the pancreas, rectum, abdominal serosa, uterus, vulva, thigh, and heart [2].

Data on PEComas arising within the gastrointestinal tract remain limited to isolate case reports [9–22]. This is the first description, to the best of our knowledge, of a case of malignant PEComa of the esophagus.

## 2. Case Report

### 2.1. Patient History

A 75-year-old Caucasian man was referred to the Department of General Surgery at the University of Padua Hospital for ingravescent dysphagia. The patient had no medical and no family history of gastroesophageal malignancies. The physical examination showed no abnormalities. Hematological and chemical studies, including tumor markers, gave normal results. A dominant polypoid esophageal mass with distal erosion, measuring 8.0 cm, was documented at EGDS (between 31 and 39 cm from the incisors). Brushing cytology was consistent with poorly differentiated small cell neoplasm and tumor cells were negative for both cytokeratins (MNF116) and chromogranin in immunohistochemical analysis performed on the cell block specimen.

**Figure 1:**
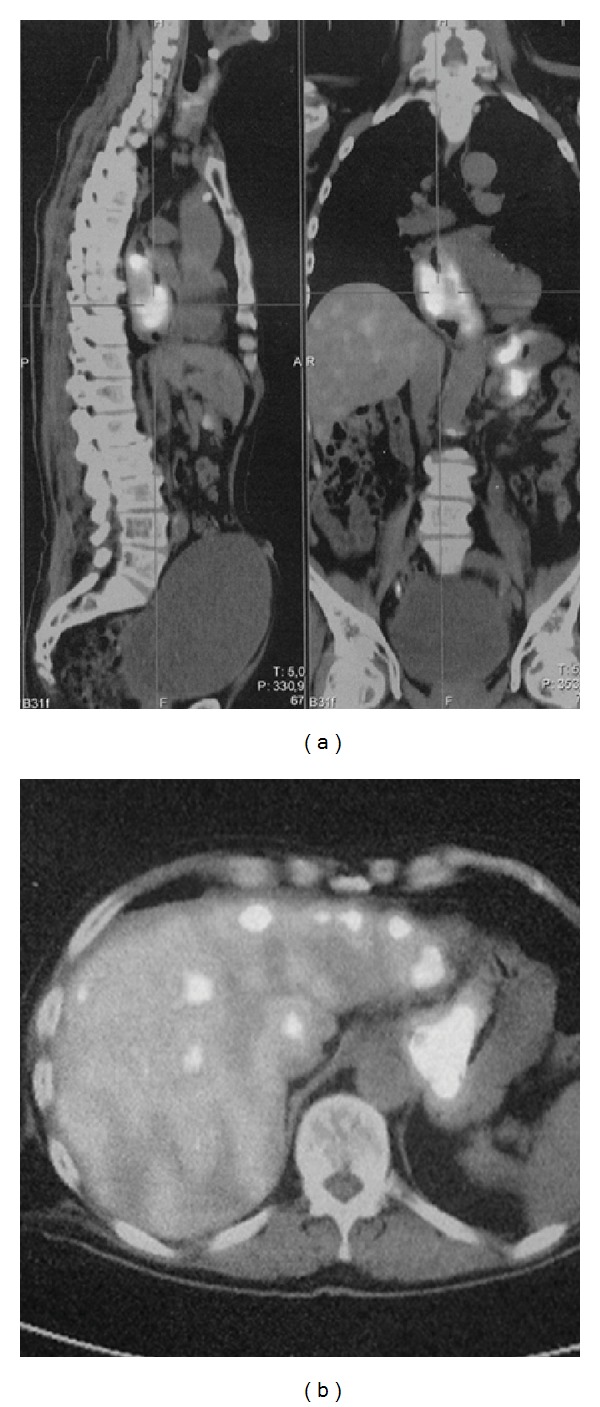
PET-TC scan showing the bulky esophageal mass (a) and the secondary diffuse liver involvement (b).

A subsequent total body CT scan confirmed the bulky lesion extending from the carina to the cardial level. Moreover, also numerous hypodense small round lesions involving the liver (extending to all the eight liver segments), and thoracoabdominal lymph nodes (pericardial, lesser curve, and celiac groups) were detected. Also a PET-CT was performed, confirming the CT data (Figure 1).

The patient underwent exploratory laparotomy, which revealed (additionally to the neoplastic liver involvement) diffuse encasing of the diaphragmatic peritoneum and of the omentum by finely scattered nodules alternating with thin, gray-white tumor rinds showing hemorrhagic variegations on the cut surface. Multiple tumor paracardial nodules were biopsied and a percutaneous transperitoneal jejunostomy for delivery of enteral nutrition was placed.

No further therapy was administered. The patient died 3 months after the initial diagnosis for a devastating course of the neoplastic disease and progressive physical deterioration. No postmortem examination was allowed.

### 2.2. Pathological Findings

**Figure 2:**
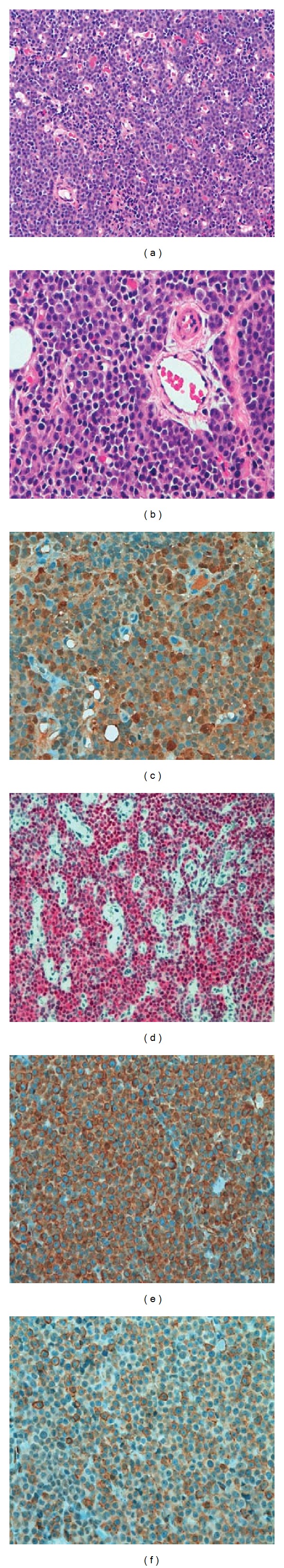
Histological and immunohistochemical features observed in the present case. (a-b) Histological evaluation of hematoxylin and eosin (H&E) stained sections showed a diffused uniform proliferation of polygonal cells, showing a round to oval nucleus. Eosinophilic cell cytoplasm was variably abundant, clear to granular. (c–f) Immunohistochemically, the tumor cells were positive for S100 (c), Mitf (d), vimentin (e), and smooth muscle actin (f). (Original magnifications ×20 and ×40.)

Histological evaluation (H&E) on the tumor biopsy samples obtained from the paracardial nodules showed a diffused proliferation of polygonal/spindle cells, characterized by a round to oval nucleus (Figures 2(a)-2(b)) with nospecific growth pattern. The cell cytoplasm was variably abundant, clear to granular. High vascular density areas were observed; tumor cells were steadily separated from capillary walls by eosinophilic bands (Figure 2(a)). Necrosis was not found and tumor exhibited three mitotic figures ×50 high power fields (HPF).

Immunohistochemistry (IHC) was performed on the Ventana automated system (Ventana Benchmark XT system; Touchstone, AZ USA) on 5 *μ*m thick sections obtained from formalin-fixed, paraffin-embedded tissue. The applied antibodies and working dilutions are listed in Table 1. Sections were lightly counterstained with hematoxylin and appropriate positive/negative controls were run concurrently. The immunoreactions were semiquantitatively scored by applying a conventional 4-tiered scale: 0 = no immunoreactions; 1 = positive immunoreactions in 33% (or fewer) of the tumor cells; 2 = positive immunoreactions in 34–65% of cells; 3 = positive immunoreactions in more than 65%. The results of immunohistochemistry are summarized in Table 1 and in Figures 2(c)–2(f). The tumor cells showed strong, diffuse positive cytoplasmic staining to HMB45, S100, vimentin and strong nuclear reactivity to microphthalmia transcription factor (Mitf). Cancer cells were also positive for HHF35, whereas were negative for desmin, CD45 (LCA), CD99, chromogranin, and cytokeratins.

## 3. Discussion

PEComa is a rare mesenchymal neoplasia belonging to a heterogeneous family of tumors, including the pulmonary “sugar” tumor and lymphangiomyomatosis and, in the abdominal cavity, the renal angiomyolipoma and the clear cell myomelanocytic tumor of the falciform ligament/ligamentum teres [2, 23]. Moreover, this group of tumors has been associated to genetic alterations of the tuberous sclerosis complex (TSC) [2–4].

Data on PEComas arising within the gastrointestinal tract remain limited to isolate case reports and 21 cases have been described so far, including 1 in the stomach, 3 in the small bowel, 12 in the colon, 1 in the appendix, and 4 in the rectum [9–22].

By the histological point of view, PEComas are composed by the proliferation of perivascular epithelioid cells (PECs). PECs are hybrid tumor cells characterized by immunoreactivity for both melanocytic and smooth muscle markers, such as HMB45, HMSA-1, MelanA/Mart1, microphthalmia transcription factor (Mitf), actin, and, less commonly, desmin; the immunoreactivity for vimentin is usually inconspicuous [3–5]. The tumors show a perivascular location, with radial arrangement around the vascular lumen of epithelioid/spindled cells, with clear to lightly granular eosinophilic cytoplasm. The pathological findings in our case were consistent with those of PEComa and immunohistochemical data further confirmed the histological diagnosis.

Several neoplasms should be considered in the differential diagnosis of this entity. In fact, in addition to epithelioid smooth muscle tumors (i.e., epithelioid leiomyosarcoma and epithelioid leiomyoma), the other important differential diagnosis of PEComa includes malignant melanoma. PEComas can be differentiated from malignant melanoma based on a negative history for melanoma, visceral location of tumor, perivascular accentuation of tumor cells, immunoreactivity for myoid markers (i.e., smooth muscle actin, muscle-specific actin, and desmin), and absence of the t(12:22) translocation [2]. Another important finding is the S100 positivity observed in melanoma; however, up to 11% of PEComas (as in our case) express S100 as well [23]. According to these data, tumors with strong and diffuse melanocytic marker positivity, and actin immunoreactivity should be designated as PEComas based on morphology, immunophenotype, and clinical history.

Even not well established, criteria for malignancy have been proposed. WHO guidelines suggest that PEComas should be regarded as malignant when they display infiltrative growth, marked hypercellularity, nuclear enlargement, hypercromasia, high mitotic activity, atypical mitotic figures, and coagulative necrosis [2]. More recently it has been reported that tumor size over 5 cm, with infiltrative growth pattern, high nuclear grade, necrosis and mitotic activity over 1/50 HPF, is significantly associated with aggressive clinical behaviour of PEComas of soft tissue and gynaecologic origin [3, 10, 22].

In conclusion, we presented, to the best of our knowledge, the first description of a case of malignant tumor which fulfilled all the morphological, immunohistochemical, and clinical criteria for a final diagnosis of malignant PEComa of the esophagus. Accurate recognition of this entity is essential because of potential misdiagnosis as other malignant tumors, especially malignant melanoma. Although these tumors are exceptional findings, they should not be excluded in the diagnostic investigation of a neoplastic lesion of the esophageal wall.

## Figures and Tables

**Table 1:** Immunohistochemical profile of the tumor.

Antigen	Clone and source	Dilution	Score values
Melanosome	HMB45 (Dako, Glostrup, Denmark)	Prediluted	++−
S100	polyclonal (Ventana, Tucson, AZ, USA)	Prediluted	++−
Vimentin	V9 (BioGenex, San Ramon, CA, USA)	1 : 100	++−
Mitf	D5 (Dako)	1 : 100	++−
Smooth muscle actin	HHF35 (Dako)	1 : 800	+−−
Desmin	D33 (Dako)	1 : 50	—
High molecular weight cytokeratins	34betaE12 (Ventana)	Prediluted	—
Low molecular weight cytokeratins	CAM 5.2 (Becton-Dickinson, Mountainview, CA, USA)	1 : 10	—
Intermediate/low-molecular-weight cytokeratins	MNF116 (Dako)	1 : 50	—
CD45 antigen	RP2/18 (Ventana)	Prediluted	—
CD99 antigen	12E7 (Dako)	Prediluted	—
Chromogranin	DAK A3 (Dako)	1 : 100	—
bcl-2 oncoprotein	124 (Dako)	1 : 50	—
CD20 antigen	L26 (Ventana)	Prediluted	—
CD3 antigen	2GV6 (Ventana)	Prediluted	—
CD138 antigen	B-A38 (Ventana)	Prediluted	—
Kappa chains	polyclonal (Ventana)	Prediluted	—
Lambda chains	polyclonal (Ventana)	Prediluted	—
PDGFRA	C-20 (Dako)	1 : 100	—
